# Penile eruptive syringoma

**DOI:** 10.11604/pamj.2017.28.268.14437

**Published:** 2017-11-28

**Authors:** Fred Bernardes Filho, Andreia De Oliveira Alves

**Affiliations:** 1 Dermatology Division, Department of Medical Clinics, Ribeirão Preto Medical School, University of São Paulo, Ribeirão Preto, Brazil; 2 Medical School, Centro Universitário Barão de Mauá, Ribeirão Preto, São Paulo, Brazil

**Keywords:** Syringoma, penile diseases, warts

## Image in medicine

A 23-year-old man presented with a six-year history of asymptomatic papular lesions on the penis. He had no history of sexually transmitted disease and reported having sought dermatologic care because he was beginning a relationship and his new girlfriend wanted to be certain that he did not have a contagious disease. Clinical examination showed multiple yellow-colored papules, 1-3 mm in size, along the dorsum and sides of the penis. Similar lesions were not present elsewhere on the body. Punch biopsies of two lesions were performed and on microscopic examination a diagnosis of syringoma was made. The patient refused treatment once the lesions were asymptomatic. Syringoma exclusively located to the penis is an extraordinary rare dermatological condition. These lesions appear as one or multiple asymptomatic papules measuring from 1 to 3 mm in diameter. Penile syringomas usually present as multiple, flesh-coloured or brownish papules on the penile shaft at puberty or in early adult life.

**Figure 1:**
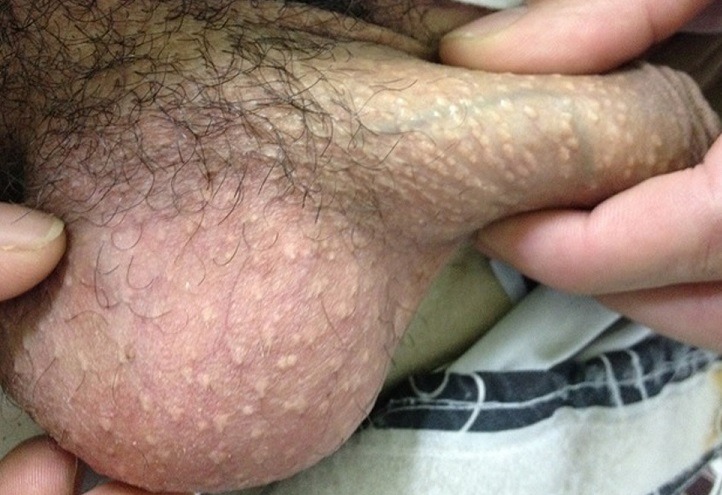
multiple 1 to 3 mm yellow-colored papules along the dorsum and sides of the penis

